# Validation of the Revised Multicultural Ideology Scale (MCI-r) in the UK

**DOI:** 10.1177/00332941221142002

**Published:** 2022-11-24

**Authors:** Katharina Lefringhausen, John W. Berry, Dmitry Grigoryev, Maria Stogianni

**Affiliations:** Department of Psychology, 3120 Heriot-Watt University, Edinburgh, UK; Center for Sociocultural Research, 68192 HSE University, Moscow, Russia; Department of Culture Studies, 7899 Tilburg University, Tilburg, The Netherlands

**Keywords:** multicultural ideology, intercultural relations, cultural diversity, integration, measures & statistic

## Abstract

As worldwide migration continues to grow, valid and reliable instruments are needed to assess the psychological processes that influence the successful management of intercultural relations in different sociopolitical contexts. In this study, we test whether the original Multicultural Ideology Scale (MCI) required a revision to remain ‘fit for purpose’ in the current culturally plural context of the UK (MCI-r). Specifically, six subscales are proposed to underlie the construct of a multicultural ideology: Cultural Maintenance, Equity/Inclusion, Social interaction, Essentialistic Boundaries, Extent of Differences, and Consequences of Diversity. With data from 300 UK nationals, we tested the psychometric properties of the MCI-r using various confirmatory factor analysis techniques to estimate the scale’s factor structure followed by convergent and discriminant validity tests. The results indicated that a 4-factor solution (Cultural Maintenance, Equity/Inclusion, Social interaction, and Consequences of Diversity) fitted the data best. All four subscales demonstrated adequate internal consistency as well as convergent and discriminant validity. All four subscales were also negatively correlated with a right-wing political orientation, whilst especially Social Interaction and Consequences of Diversity were positively associated with intergroup contact frequency across domains (work, family and friends and/or acquaintances). Finally, UK participants with personal migratory experiences reported a stronger belief in positive consequences of multiculturalism and more support for Social Interactions between members of different ethnic groups. Overall, findings provide first insights into the applicability of the MCI-r as a reliable and valid tool for the assessment of multiculturalism within the present UK context.

## Introduction

Global immigration is increasing, with 281 million people living outside their country of birth or citizenship in 2020 (UN, 2021). This trend is further driven by the current conflicts such as the wars in Afghanistan, Syria and Ukraine as well as the environmental threats imposed by climate change to people’s habitats. The resulting dynamics between this ongoing growing number of more diverse ethnic and national origins (entailing multiple languages and religions) creates new patterns of inequality, prejudice, and a general new experience of intergroup contacts (Vertovec, 2019). For example, the Second European Union Minorities and Discrimination Survey (European Union Agency for Fundamental Rights, 2017) indicated a continuous high level of people experiencing discrimination, as well as physical violence and harassment motivated by hatred due to their ethnicity, race or country of origin. This gives reason to re-examine past approaches and their operationalization on how to understand and manage intercultural relations in current culturally plural societies.

Throughout history, many societies have attempted to manage these intercultural relations. For most, the strategy was to either assimilate or segregate the non-dominant group members (e.g., immigrants) to the dominant-/majority group culture (Berry, 1984; Gordon, 1964). Recognising that these two approaches were not working, the Government of Canada (1971) introduced a policy of multiculturalism that sought to improve intercultural relations by proposing two initiatives: supporting the maintenance of heritage cultures, and by promoting social interactions and sharing of these cultures among all residents. In response to this policy, Berry et al. (1977) were the first to carry out research on the study of multiculturalism as a psychological concept. They sought to describe various ways that different cultural groups in a culturally diverse society can relate to each other. They developed the concept of *multicultural ideology* that incorporates a preference for both heritage culture maintenance, and for contact and social participation of all ethnocultural groups. Thus, rather than describing only the extent of support for cultural diversity in a society, multicultural ideology also assesses peoples’ preferences for intercultural contact, resulting in complex set of attitudes (ideologies) about how to best manage diversity as a way to improve mutual intergroup relationships. As a result, MCI is more than a ‘diversity ideology’ (Rattan & Ambady, 2013) but is an ‘intercultural ideology’, one that examines preferences for different relationships among diverse cultural groups. Many other intercultural ideologies have since been proposed and have been categorised according to the issues that they prioritise (Grigoryev & Berry, 2021).

To assess multiculturalism as a personally held intergroup ideology, Berry et al. (1977) and Berry and Kalin (1995) developed the Multicultural Ideology Scale (MCI). Since then, the complex dynamics related to changing contemporary diversity has introduced potentially new or different meanings of multiculturalism; this renders it necessary to test the applicability of the original MCI across various sociocultural contexts. Thus, as part of an international research project which aims to test the psychometric properties of a revised MCI (MCI-r) in different language versions and sociocultural contexts, we examined the scale within the current UK context.

## Conceptualization of Multiculturalism as an Intergroup Ideology

Berry et al. (1977) and Berry (2017) proposed four ways of managing intercultural relations for dominant group-members based on the two issues in the Canadian policy: (1) the maintenance of heritage cultures; (2) the equitable social participation of all cultural group-members in the larger society. The essence of this view is that maintaining and sharing of cultures among all cultural communities is a public and personal ‘good’. Opposite views, such as eliminating the core qualities of a group, or of establishing boundaries between them, do not belong in the domain of multiculturalism. These contrasting views result in a multiculturalism ideology that values diversity and social participation at one end of the ideological spectrum and segregationism (i.e., forced separation between cultural groups), assimilationism or melting pot (i.e., non-dominant cultural groups should reject their heritage culture to fully endorse the mainstream culture) and exclusionism (i.e., rejecting non-dominant cultural groups participation in the mainstream culture as well as their heritage culture maintenance) on the other end (Berry, 2003).

Notably, other intergroup ideologies have been proposed that compete with the conceptualization of multiculturalism as proposed by Berry et al., (1977) and Grigoryev and Berry, 2021. For example, interculturalism, like multiculturalism, “aims to value both cultural diversity and the full participation of non-dominant groups in the larger society” (p. 13). However, interculturalism also focusses on prioritizing the culture of majority members in a given society, thus favoring hierarchical rather than egalitarian pluralism in contrast to multiculturalism (Scott & Safdar, 2017). Indeed, based on the Canadian policy of multiculturalism, a multicultural ideology consists of a combination of three issues (Berry et al., 1977): the right of ethnic minority groups to maintain their heritage cultures (*Cultural Maintenance*); but also full social participation and thus, a balanced power status of all ethnocultural groups in the larger society (*Equity/Inclusion*); as well as social interactions between majority and minority group members (*Social Interaction*).

When multiculturalism is endorsed by majority group-members, minority members have the option to follow an integration acculturation strategy – maintaining their heritage culture whilst participating in the larger society – which has been repeatedly demonstrated to be correlated with beneficial psychological and sociocultural outcomes (Berry et al., 2022; Bierwiaczonek & Kunst, 2021; Stogianni, Bender et al., 2021). Moreover, endorsing multiculturalism reduces both explicit and implicit prejudice towards minority members (see meta-analysis by Whitley & Webster, 2019), reduces stereotyping and discrimination, whilst fostering pro-diversity policy support (see meta-analysis by Leslie et al., 2020). Finally, a multicultural ideology can even create a favorable social context for positive self-esteem for both minority and majority members (Verkuyten, 2009). Taken together, current research indicates strong benefits for dominant group members and their intergroup relationships when endorsing a multicultural ideology.

## Operationalization of Multiculturalism

In line with Berry et al.’s (1977) and Berry and Kalin (1995) conceptualization of multiculturalism as an intergroup ideology, the MCI was created as a brief measure to assess the domains of Cultural Maintenance, Equity/Inclusion and Social Interaction. To date, adapted versions of the scale have been used in various culturally plural societies (Berry, 2017). Within majority member samples, several studies using the MCI and its adapted versions provided evidence for its unidimensional structure, with a preference for multiculturalism at one end of the spectrum and less welcoming ideologies at the other (Breugelmans & van de Vijver, 2004; Schalk-Soekar & van de Vijver, 2008; van de Vijver et al., 2008). More recently, however, a two-factor solution was reported within a Luxemburg sample (Stogianni et al., 2021c), where residents from 170 countries make cultural diversity a daily lived reality (Le Gouvernment Du Grand-Duché Luxembourg, 2021).

In general, the MCI’s conceptualization and operationalization have been evaluated over the years; that is, although Cultural Maintenance and Equity/Inclusion were still regarded as core elements of a multicultural ideology, other aspects have been more emphasized or added (Stogianni et al., 2021c, 2021) or even alternative intergroup ideologies proposed (e.g., interculturalism; Verkuyten et al., 2020). With regard to re-conceptualizing a multicultural ideology, Grigoryev and Berry (2021) noted the need to explore the potential incorporation of: (1) recognition of cultural differences between ethnic groups and that they should remain distinct (*Extent of Differences*); (2) the potential value of cultural diversity in a society, which may enable to solve new problems more effectively (*Consequences of Diversity*); and finally, (3) de-emphasizing essentialistic beliefs about ethnic group membership in that culture (*Essentialistic Boundaries).* These issues have been addressed by Breugelmans and van de Vijver (2004), Guimond et al. (2014), Moftizadeh et al. (2021) and Stuart and Ward (2019). For example, Consequences of Diversity was explored as a distinct domain of multiculturalism by Breugelmans and van de Vijver (2004) within a Dutch native sample (“Domain of multiculturalism in the Netherlands”, p. 407), reporting that participants were more likely to support multiculturalism when measured as minority members societal participation rather than in terms of embracing the consequences of multiculturalism. Thus, the present study aimed to address this need to include such newly identified potential components of multiculturalism by testing the psychometric properties of its six proposed subscales.

## The Present Study

The present study tested the MCI-r which was developed in English and incorporates six subscales that refer to three conceptual attitude dimensions: one old dimension in form of the integration of immigrant groups (Cultural Maintenance, Equity/Inclusion and Social Interaction) and two new dimensions, which refer to the consequences of cultural diversity and dealing with cultural differences between groups (Extent of Differences, Consequences of Diversity, and Essentialistic Boundaries; see Stogianni, Berry et al., (2021) for additional details).

Notably, the MCI-r asks participants about their beliefs with respect to an ideal or what ‘should be’ regarding managing a culturally plural society in which they live (i.e., prescriptive aspect) rather than their actual experiences or observations with regard to living in such a society (i.e., descriptive aspect). For example, Stuart and Ward (2019; see also Guimond et al., 2014) follow a descriptive approach, by assessing individuals’ perceptions of how multiculturalism is practiced and endorsed (or not) by society, its institutions and members. Thus, normative multiculturalism indicates how individuals perceive multicultural policies and practices, multicultural ideology and contact with diversity ‘as is’ in their society. Multicultural Ideology as defined by Berry et al. (1977) and as assessed with the MCI-r does not assess such descriptive norms of multiculturalism; nor is it related to the concept of injunctive norms (i.e., perception of whether one’s in-group approves/disapproves of certain attitudes or behaviours; Cialdini et al., 1990). In contrast, MCI-r assesses people’s individual preferences for contact with and management of cultural diversity in their society.

Stogianni, Berry et al. (2021) tested the MCI-r first in German language and in two representative community samples in Germany and Luxembourg which differ in their demographic composition and immigrant integration policies. Their results demonstrated a four-factor solution across samples, including the dimensions Cultural Maintenance, Equity/Inclusion, Social Interaction, and Consequences of Diversity, while the dimensions Essentialistic Boundaries and Extent of Differences revealed poor psychometric properties. The four subscales also represented distinct but interrelated dimensions of multicultural ideology, contrasting previous work on the original scale that reported a unidimensional structure (e.g., Berry & Kalin, 1995). Notably, however, the findings in Stogianni, Berry et al. (2021) also revealed non-invariance of the scale across the German and Luxembourg samples. In other words, participants seemed to interpret some MCI-r items differently across countries as a potential result from their different immigrant integration policies with Luxembourg being a stronger advocate for cultural diversity with more supportive integration policies relative to Germany (Migrant Integration Policy Index, 2020).

Looking at the UK, an estimated 14.5% of its population was born abroad of which 36% were born in the EU (mostly from Poland) whilst most of non-EU foreign-born population come from India (Vargas-Silva & Rienzo, 2022). However, following the referendum vote in 2016 which resulted in the UK leaving the European Union (referred to as Brexit), immigration policies for EU-immigrants have become more restrictive, with the government aiming to lower the country’s general immigration influx (Sumption & Kierans, 2021). Nevertheless, the country’s integration policy reflects that of Germany by providing immigrants with basic rights and equal opportunities, yet without fostering their recognition as citizens with a secure future in the country (Migrant Integration Policy Index, 2020). Indeed, the UK is by now known to create a ‘hostile environment’ for ethnic minorities via its immigration and integration policies (Griffiths & Yeo, 2021). For example, directly after the Brexit vote, England and Wales witnessed a severe increase in racially motivated hate crimes compared with the same month the previous year, which lasted almost a year after the referendum (Bulman, 2017).

Yet, whilst ‘immigration’ was perceived to be the ‘most important issue’ facing the British public in 2015–2016 (48%), this view had dropped drastically by November 2019 (13%; Blinder & Richards, 2020). A similar reduction was recorded when asking British citizens about immigration influx – that is, in 2013, 77% of surveyed participants (*N* = 3076, representative sample) favoured a reduction in numbers whereas in 2019 this number dropped to 44% (Blinder & Richards, 2020). Meanwhile, 64% of respondents of a recent British Social Attitudes survey (Clery et al., 2021) reported that immigration enriches the UK’s culture, yet with those describing themselves as strong ‘Leavers’ showing much less agreement with this statement (30%) than those describing themselves as strong ‘Remainers’ (83%).

With data from this current sociocultural context, we investigated the factorial structure of the MCI-r. Additionally, we explored the dimensions’ intercorrelations as well as estimated indicators of reliability, discriminant and convergent validity. To gain further insights into the scale’s convergent validity, we also explored the MCI-r’s associations with theoretically distinct but related constructs, expecting higher endorsement of multiculturalism to relate to a liberal political orientation (e.g., Rydgren, 2007), a higher level of intergroup contact frequency, migratory experiences and background (e.g., Maddux et al., 2021; Visintin et al., 2019).

## Method

### Participants

We collected data from 300 participants who fulfilled the following requirements: were born in the UK, had British citizenship, their first language was English and they were living in the UK at the time of the survey. The majority of respondents was women, born in England, self-identified as white, completed secondary school, held a completed academic qualification and had no migratory experiences or parents with a migratory background (see Table 1). The age ranged between 18 and 74 years and 48% described their socioeconomic status as “about the same” in comparison to other people living in the UK.

**Table 1.** Descriptive Statistics of the Sample (*N* = 300).

Demographics		*n*	%
Gender	Woman	199	66.3
	Man	97	32.3
	Other^a^	4	1.3
Country of birth	England	260	86.7
	Other	36	12.0
	Missing	4	1.3
Ethnicity	White (English/Welsh/Scottish/Northern Irish/British; Irish; Gypsy or Irish Traveller; any other White background)	263	87.7
	Other	27	12.3
Schooling	Completed secondary school	155	51.7
	Completed community college	90	30.0
	Other completed schooling	54	18.3
Qualification	No academic level	51	17.0
	Current student	35	11.7
	Completed BA degree	96	32.0
	Completed postgraduate degree or PhD	91	30.3
	Other completed academic degrees	27	9.0
Migratory experience	Not applicable	192	64.0
	Having lived abroad for less/more than 1 year	108	36.0
Migratory background	None	232	77.3
	One or both parents were born abroad	68	22.7
Age	M (*SD*)	36.34 (13.79)	
Perceived SES	M (*SD*)	3.31 (0.75)	
Perceived COVID-19 impact	M *(SD)*	3.60 (1.32)	

^a^One non-binary, and one transwoman.

### Measures

#### The Multicultural Ideology Scale-revised

Using 24 items (8 negatively worded), the MCI-r asked participants on a 5-point Likert scale (1 = *strongly disagree*, 5 = *strongly agree*) about their personal views across six dimensions of multiculturalism (for item development see Stogianni, Berry et al., 2021): *Cultural Maintenance* (CM; e.g., “It would be good to see all ethnic groups in the UK retain their cultures”), *Social Interaction* (SI; e.g., “I think that immigrants and people in the UK should seek more contact with one another”), *Equity/Inclusion* (EQ; e.g., “I think that immigrants in the UK should have equal rights as people already living here”), *Extent of Differences* (DI; e.g., “All cultures should have their own distinct traditions and perspectives”), *Consequences of Diversity* (CD; e.g., “A society which has a variety of ethnic groups is more able to tackle new problems as they occur.”), *Essentialistic Boundaries* (EB; e.g., “Racial and ethnic group memberships do not matter very much to who we really are”). Higher scores indicated more endorsement of multicultural ideology.

#### Additional Variables

We measured participants’ political orientation with 1-item: “Some people talk about left, right or centre to describe parties and politicians. With this in mind, where would you place yourself from 1 (left) to 10 (right)?”. Higher scores reflected a political orientation towards the right, representing ideas such as social hierarchy and nationalism. We also asked participants, “In which of these domains [work, family and friends and/or acquaintances] do you have contact with current immigrants or those with an immigration background?” Answers were recorded on a 3-point Likert scale (1 = *never*, 2 = *sometimes*, 3 = *regularly*). Higher scores indicated more intergroup contact. Given that data was collected during the Covid 19 pandemic, we also included one item to assess to what extent participants perceived that the pandemic had in general a negative impact on their well-being on a six point-Likert scale (1 = *not at all*, 6 = *very much*).

### Procedure

An online version of the survey was developed in English using Qualtrics. Data was collected via the participant recruitment platform Prolific. Participants were paid £1.25 upon survey completion. Ethical approval was received on the fourth of August 2020 and data was collected in January 2021. First participants were asked to provide their demographic information (including their political orientation, intergroup contact frequency, migratory experiences and background) followed by the MCI-r items in randomized order.

## Results

### Analysis Plan

Confirmatory factor analyses (CFA) were performed via JASP Team (2018), using the robust maximum likelihood estimator. We employed CFA given its purpose to test pre-defined models or factor structures (Suhr, 2006). Indeed, we expected that the MCI-r shows a six-factor structure reflecting the dimensions meant to capture multicultural ideology as outlined in our literature review. Similar to Stogianni, Berry et al. (2021), we therefore followed a ‘transport and test’ methodological approach in which the applicability of a hypothesised model for one sociocultural context is tested in another (Matsumoto & Juang, 2016). In the case of Stogianni, Berry et al. (2021), only four factors were supported across contexts. In so doing, we decided not to conduct exploratory factor analysis (EFA) and CFA for the same sample. Firstly, EFA should mainly be used to explore possible underlying factor structures without imposing a hypothesized structure on the outcome (Child, 1990). Secondly, Fokkema and Greiff (2017) have shown that combining both analyses methods leads to over fitting of the tested model, resulting in inflated estimates of model fit, parameter estimates, and test statistics.

First, we tested for first-order CFA models in form of (a) a 1-factor model (unidimensional) with all MCI-r items loading on a single latent variable, (b) a 6-factor model with MCI-r items loading onto their respective latent variable, allowing these latent variables to be correlated, (c) a 5-factor model after excluding one subscale with no significant correlations, and (d) a 4-factor model after excluding a second subscale with (partially) no significant correlations. This was followed by second-order and bifactor CFA models. As the chi-square statistic, which should be non-significant, is sensitive to sample size, we used multiple fit indices to determine the model fit (Kline, 2011): the comparative fit index (CFI; should be greater than 0.90), the root-mean-square error approximation (RMSEA; should be smaller than 0.05 or less than 0.08 for an acceptable fit), and the standardized root-mean-square residual (SRMR; should be 0.08 or less).

Additional analyses were performed to examine the psychometric properties - that is, we inspected Cronbach’s alpha and McDonald’s omega coefficients to inform about the instrument’s internal consistency (reliability). Convergent validity was estimated with the average variance extracted (AVE) and discriminant validity was inspected via the Heterotrait-Monotrait Ratio of Correlations (HTMT). Moreover, we explored correlations between the final MCI-r dimensions with participants’ political orientation and their level of intergroup contact. Additionally, we inspected whether participants varied in their endorsement of the MCI-r dimensions with regard to their migratory background and experiences.

### Measurement Model

Table 2 shows the goodness-of-fit indices for each model. Because past studies using the MCI provided evidence for its unidimensional structure, we first tested a 1-factor solution, with all items loading on one latent construct. This model indicted a poor fit to the data and seven items showed factor loadings lower than .30 of which four were not significant. Next, we tested a 6-factor model, (with correlated factors; e.g., Brown, 2015) including all dimensions from the old MCI (CM, SI, EQ) and the revised MCI: EB, CD, and DI. The six-factor structure was not supported with all fit indices not meeting their recommended cut-off values (see Table 2). Notably, the factors DI and partially the factor EB had no significant correlations with the other four MCI-r factors. Nevertheless, most components had relatively high factor loadings ranging from 0.50 to 0.97.

**Table 2.** Summary of Model Fit indices for the Estimated Measurement Models (*N* = 300).

Model		Model Fit				Model Comparison	
		χ^2^ (*df*)	CFI	RMSEA [90% CI]	SRMR		
1	Single-factor CFA model	1587.283* (252)	0.563	0.133 [0.127, 0.139]	0.117		
2	Six-factor CFA model with correlated grouping factors	632.440*(237)	0.871	0.075 [0.068, 0.082]	0.079		
3	Single-factor with DI dropped	1094.649* (170)	0.648	0.135 [0.127, 0.142]	0.106		
4	Five-factor CFA model with DI dropped and correlated grouping factors	435.521*(160)	0.895	**0.076 [0.067, 0.084]**	**0.068**		
5	Single-factor CFA model with DI and EB dropped	658.590*(104)	0.750	0.133 [0.124, 0.143]	**0.078**		
6	Four-factor CFA model with DI and EB dropped as well as correlated grouping factors	319.203*(98)	**0.900**	0.087 [0.076, 0.097]	**0.051**	Model 4 versus Model 6	**Δ**χ^2^ (62) = 116.32, *p* < .001
7	Second-order factor CFA model with DI and EB dropped	320.058*(100)	**0.901**	0.086 [0.075, 0.096]	**0.048**	Model 6 versus Model 7	**Δ**χ^2^ (2) = 0.85, *p* > .050
8	Bifactor CFA model with DI and EB dropped	Did not converge					
9	Bifactor CFA model with DI and EB dropped as well as correlated grouping factor	Did not converge					

*Note.* df = Degrees of freedom; CFI = Comparative fit index; RMSEA = Root-mean-square error of approximation; SRMR = Standardized root mean square residual; In bold: meets the model fit criterion.

*p < .001.

We then tested for a correlated 5-factor model, dropping DI. Although the model fit improved with regard to the RMSEA and SRMS values, an acceptable CFI level could still not be reached (see Table 2). Notably, again, EB showed only two significant covariance pathways (with CM, *p* = .010, and CD, *p* = .019). Thus, we then tested a correlated 4-factor model, excluding DI and EB. This model showed an acceptable fit with regard to the CFI and SRMR values. All factor loadings were significant (*p* < .001) and above 0.57 (see Table 3), as well as all 4-factors were significantly inter-correlated with values ranging from 0.60 to 0.91 (*p* < .001). Moreover, in comparison to a 5-factor model, a 4-factor model showed a significant reduction in the chi-square value (see Table 2).

**Table 3.** Reliability Coefficients and Factor Loadings for the Four-Factor Confirmatory Factor Analyses Model (*N* = 300).

Items		α/ω	Loading s
Cultural Maintenance (CM)		0.87/0.86	
1	It would be good to see all ethnic groups in the UK retain their cultures		0.69
2	It is best for the UK if all ethnic groups forgot their cultural backgrounds as soon as possible (R)		0.61
3	People who come to the UK should change their behaviour to be more similar to the local population (R)		0.63
4	We should help ethnic and racial minorities preserve their cultural heritages in the UK		0.81
Equity/Inclusion (EQ)		0.87/0.86	
5	I think that immigrants in the UK should have equal rights as people already living here		0.97
6	I think that immigrants in the UK should have fewer rights than those who were born here (R)		0.90
7	I think that immigrants in the UK should enjoy the same freedoms as those who were born here		0.83
8	I think that immigrants in the UK should not have the same freedoms as those already living here (R)		0.76
Social Interaction (SI)		0.77/0.79	
9	If members of ethnic groups want to keep their own culture, they should keep it to themselves and not bother other people in this country (R)		0.85
10	We should do more to learn about the customs and heritage of different ethnic and cultural groups in this country		0.87
11	I do not like being on a bus or a train in which there are many people of other cultures (R)		0.58
12	I think that immigrants and people in the UK should seek more contact with one another		0.57
Consequences of Diversity (CD)		0.74/0.74	
13	Having a lot of different cultural groups in the UK makes it difficult to solve problems in our society (R)		0.85
14	The unity of this country is weakened by ethnic groups sticking to their old ways (R)		0.76
15	A society which has a variety of ethnic groups is more able to tackle new problems as they occur		0.63
16	I feel at ease when I am in a city with many immigrants		0.73

*Note.* Factor loadings were all significant (*p* < .001). R: items are reverse coded.

On the basis of a correlated 4-factor solution, we then tested whether a second-order or bifactorial model would show an even better fit with the data. The second-order model (Thurstone, 1944) hypothesizes at least one superordinate factor and multiple first-order factors upon which a specified sub-group of items load. This second-order factor – here, multicultural ideology – explicitly models the shared variance between first-order factors (i.e., CM, EQ, SI, and CD). Thus, the first-order factors are uncorrelated with one another and each one mediates the relationship between the second-order factor and the observed variables (Markon, 2019). For our sample, a second-order model did not indicate a significantly better fit to the data than a correlated 4-factor solution (see Table 2).

The bifactor model (Holzinger & Swineford, 1937) or a hierarchical model (Markon, 2019) hypothesizes a general factor – here, multicultural ideology – which loads directly onto all of the observed variables in the model. Additionally, grouping factors (i.e., CM, EQ, SI, and CD) load onto sub-groups of the same set of observed variables. These grouping factors are hypothesized to be uncorrelated with the general factor, whereas grouping factors themselves can be either correlated or uncorrelated. For our sample, a bi-factorial model with un- and correlated grouping factors did not converge.

### Convergent and Discriminant Analyses

Convergent validity indicates that a new scale is related to other variables that measure the same underlying construct. This can be estimated via the AVE criterion which refers to the average amount of variance explained by a construct in its indicator variables relative to the overall variance of its indicators (Fornell & Larcker, 1981; Henseler et al., 2015). Values higher than .50 support convergent validity, whereas values below 0.50 indicate that the variance due to measurement error is larger than the variance captured by the construct (i.e., low convergent validity; Grigoryev et al., 2020). Among the final four subscales, EQ (AVE = 0.75), SI (AVE = 0.54), and CD (AVE = 0.56) demonstrated sufficient convergent validity whereas CM was just below the threshold (AVE = 0.47).

To test for discriminant validity, we calculated the HTMT: the average of the correlations of indicators across constructs measuring different phenomena (i.e., heterotrait-heteromethod correlations) relative to the average of the correlations of indicators within the same construct (the monotrait-heteromethod correlations; Nunnally, 1978). Lower HTMT values than a conservative threshold of .85 or a more lenient threshold of 0.90 indicate support for discriminant validity (Henseler et al., 2015; Kline, 2011). For our UK sample, discriminant validity was supported for three subscales in respect to the more conservative threshold (CM, EQ, and CD) and for all four subscales in respect of the more lenient threshold (see Table 4).

**Table 4.** Discriminant Validity Heterotrait-Monotrait Ratio of Correlations Coefficients between the MCI-r Subscales (*N* = 300).

	1	2	3	4
1. Cultural maintenance	—			
2. Equity/inclusion	0.59	—		
3. Social interaction	**0.89**	0.70	—	
4. Consequences of diversity	0.82	0.65	**0.87**	—

*Note.* The HTMT values larger than 0.85 are bold.

### Additional Analyses

We explored the relationships between the MCI-r dimensions with participants’ political orientation, and intergroup contact frequency to further explore the dimensions’ convergent validity. Because EQ, SI and intergroup contact at work were not normally distributed, we conducted Spearman’s Rank Order Correlation (see Table 5). Results showed that all four subscales of the MCI-r were negatively related with a stronger political orientation towards the right with medium to large effect sizes. Moreover, especially SI and CD were positively related, yet weakly, with all three intergroup contact indicators.

**Table 5.** Spearman’s Correlations Between MCI-r Subscales and Demographic Variables (*N* = 300).

		1	2	3	4	5	6	7	8
MCI-r Subscales									
1	Cultural Maintenance (CM)	—							
2	Equity/Inclusion (EQ)	0.47***	—						
3	Social Interaction (SI)	0.66***	0.54***	—					
4	Consequences of Diversity (CD)	0.59***	0.50***	0.63***	—				
Additional Variables									
5	Political Orientation	−0.49***	−0.44***	−0.48***	−0.52***	—			
6	Intergroup Contact - Work	0.03	0.01	0.20***	0.14*	−0.10	—		
7	Intergroup Contact - Family	0.14*	0.06	0.15*	0.22***	−0.05	0.16**	—	
8	Intergroup Contact - Friends	0.11	0.12	0.20***	0.21***	−0.05	0.36***	0.36***	—
Scale range		1–5	1–5	1–5	1–5	1–10	1–3	1–3	1–3
M (*SD*)		3.90 (0.79)	4.28 (0.93)	4.14 (0.81)	3.70 (0.86)	3.92 (2.03)	2.20 (0.76)	1.56 (0.76)	2.12 (0.66)

*Note.* *p* < .05*; *p* < .01**; *p* < .001***.

Finally, we inspected whether the endorsement of the MCI-r dimensions was different across participants’ migratory experiences (i.e., having lived abroad) and background (i.e., one or both parents were born abroad). Independent-samples *t*-tests for CD and CM revealed one significant difference across groups with a large effect size: those with migratory experiences perceived more positive consequences due to cultural diversity (*M* = 3.83, *SD* = 0.87) than those without such experiences (*M* = 3.62, *SD* = 0.85), *t* (298) = −2.04, *p* = .042, *d* = 0.24. Inspecting the same group differences across EQ and SI using a Mann-Whintey U tests showed that SI significantly varied across migratory experiences (U = 8177, *p* = .002, *d* = 0.18). Those having no such experiences reported lower scores on SI (*Md* = 4.25) than those with migratory experiences (*Md* = 4.50).^1^

## Discussion

Overall, the findings are in line with the three core dimensions of multicultural ideology by supporting the integration of immigrant groups (Cultural Maintenance, Equity/Inclusion and Social Interaction) and extending it only by considering the consequences of cultural diversity (Consequences of Diversity). In other words, for our UK sample, Essentialistic Boundaries and Extent of Differences did not constitute meaningful domains of multiculturalism; instead, its original conceptualization in form of a belief in the value of cultural diversity, and of social contact and inclusion (Berry et al., 1977) was supported.

Specifically, this four-factor model relates to two underlying domains of multiculturalism: a contact domain, consisting of Social Interaction and Consequences of Diversity; and a group status domain, consisting of Cultural Maintenance and Equity/Inclusion. This can be interpreted along two of the three proposed issues concerning the process of acculturation (Berry, 1980; Berry et al., 2022, p. 1018): contact-participation or “the degree to which there is a desire to engage in daily interactions with other groups in the larger society, including both dominant and non-dominant one(s)”; as well as group status or the “relative power of the groups in contact to choose their preferred way of engaging each other”. This result echoes Stogianni, Berry et al.’s (2021) findings in that a multicultural ideology has to be understood as more than the accepted presence of multiple cultures in a society (i.e., a ‘diversity ideology’). Consequently, we suggest that future versions of MCI-r should drop the items for Essentialistic Boundaries and Extent of Differences all together.

Moreover, the four-factor solution showed robust psychometric properties, similar to the findings by Stogianni, Berry et al. (2021) for their German and Luxembourg samples. By contrast, our findings differ from past work that indicated a unidimensional structure of a multicultural ideology (e.g., Berry & Kalin, 1995; van de Vijver et al., 2008). Yet, the emergence of a more complex structure of the meaning of multiculturalism was to be expected given our addition of more current understandings of this phenomenon to the revised scale. Consequently, our results add to the discussion of the applicability of the MCI-r within socioculturally and politically different plural societies.

Additionally, and as expected, UK participants scoring high on the four subscales of a multicultural ideology also indicated a liberal political orientation (Rydgren, 2007) as well as high levels in intergroup contact frequency (Visintin et al., 2019). Regarding the latter, however, positive relationships were only found for the subscales Social Interaction (SI) and Consequences of Diversity (CD) whereas no significant correlations were revealed for Cultural Maintenance (CM) and Equity/Inclusion (EQ). In other words, the extent to which UK participants have contact with immigrants who may be family members, friends or colleagues at work has no relation to the extent to which they expect that said immigrants can maintain their heritage culture or have equal rights to UK citizens. This finding may be the result of participants thinking of different types of ethnic minority groups. For example, British citizens are more in favour of highly skilled immigrants than low skilled immigrants, no matter their skin colour or religion (Fernández-Reino, 2021). Indeed, some participants may have thought of contact with devalued ethnic minority members, and thus preferring them not to have the option to maintain their heritage cultures or be granted equal rights to UK nationals. Alternatively, CM and EQ rather than SI and CD indicate a preference for change to the dominant status quo of the majority group and its way of life, indicating a group status domain of multiculturalism. Thus, the level of intergroup contact may not be a relevant driver for the acceptance or rejection of CM and EQ, but rather participants’ belief in whether this change is already in motion rather than being recently induced by minority groups’ presence; indeed, the latter named would abruptly disrupt the stability and status-quo of the majority group and their way of life (i.e., theory of cultural inertia; Zárate et al., 2012).

Meanwhile, UK participants who had migratory experiences (i.e., have lived less/more than 1 year abroad) reported a stronger belief in the positive consequences of multiculturalism (CD) and support for intergroup contact (SI) whereas no such differences were found across groups with different migratory background (i.e., one or both parents were born abroad). Thus, having had intergroup contact abroad or ‘at home’ seems to be vital for the acknowledgement of multiculturalism as a beneficial factor to society as well as regarding intergroup contact as desirable (e.g., Maddux et al., 2021).

Notably, we are aware that data was collected during a winter peak of COVID-19 cases in the UK (Gov.uk, 2022) which could further impact participants’ endorsement of a multicultural ideology. For example, Hartman et al. (2021) found that during the initial phase of strict lockdown in the UK and Ireland, high levels of perceived threat due to COVID-19 fostered the positive relationship between right-wing authoritarianism with nationalism and anti-immigrant attitudes. However, a correlation between our MCI-r dimensions and our COVID-19 impact variable revealed no significant relationships.

## Limitations, Future Research and Conclusions

The present study is not without its limitations. We collected data using the platform Prolific; yet to what extent it provides high quality data (e.g. in terms of authenticity and attention) is currently being debated (Litman et al., 2021; Peer et al., 2021). Poor data quality impacts the reliability of research findings as it hinders test statistics to optimize the signal to statistical noise ratio, and thus, to achieve the best representation of the phenomena studied (Buchanan & Scofield, 2018). In the present study, this could explain the poor fit of our proposed six-factor model of multiculturalism. Moreover, the items assessing Essentialistic Boundaries and Extent of Differences may have not adequately captured both concepts. Future research should therefore replicate our work using a combination of data quality checks (e.g., page response times, click counts, and attention checks) followed by sensitivity analyses (Buchanan & Scofield, 2018) as well as potential re-wording of the Essentialistic Boundaries and Extent of Differences subscales. Alternatively, the lack of a six-factor model fit may be due to different understandings of multiculturalism and its relevant aspects across sociocultural contexts. For example, Essentialistic Boundaries and Extent of Differences are discussed as problematic aspects of multiculturalism, whereas others propose multiculturalism to be their remedies (Kymlicka, 2014). Thus, future research should provide insights on potential configural or metric invariances of the scale across sociocultural contexts and understandings of multiculturalism.

In so doing, future research should also consider the unique and contradictory context of the UK and its citizens in more depth – that is, the 2016 EU referendum outcome is shaping UK’s present immigration policies whilst some voters have developed a strong attachment to being a ‘Remainer’ or a ‘Leaver’ (Hobolt et al., 2021). These policy and identity changes may impact citizens support for a multicultural ideology. Indeed, as reported by the recent British Social Attitudes survey (Clery et al., 2021), those describing themselves as ‘Leavers’ were less likely to view immigration as an enrichment for the UK relative to those describing themselves as ‘Remainers’, indicating potential variety across the Consequences of Diversity dimension of the MCI-r. Moreover, geographic differences could also be expected given that the Brexit vote divided the UK into England (especially the West Midlands) and Wales supporting Leave and Northern Ireland and Scotland (but also London) supporting Remain (BBC, 2021).

Finally, to gain a better understanding of their conceptual and predictive differences, future research could compare the predictive power of the MCI-r dimensions regarding people’s intergroup behaviours and attitudes with related concepts such as normative multiculturalism (Stuart & Ward, 2019) and majority members’ proximal-acculturation. The first named exploration could be guided by Ward et al.’s (2018) integrative framework for the psychological study of multiculturalism. The second named refers to acculturation strategies followed by majority members *themselves.* In other words, the extent to which they adopt immigrants’ cultures and/or maintain their national culture may have implications on how intergroup contact is experienced, and thus, whether Cultural Maintenance and shared power (Equity/Inclusion) is regarded as favorable (e.g., Lefringhausen et al., 2021). Indeed, the support for Equity/Inclusion and acceptance of immigrants’ heritage culture maintenance seems to require a more profound cognitive shift in majority members and their willingness to share the onus of responsibility of cultural change in a shared multicultural society (e.g., Grigoryev et al., 2020; Nortio et al., 2020).

In sum, the present study revealed the applicability of the MCI-r to better understand a new and expanded meaning of multiculturalism within a current UK context. In so doing, we demonstrated the need to revise and test instruments employed to explore societies’ experiences of intergroup contacts, inequality, and prejudice. The MCI-r constitutes a promising tool to fulfill this need, allowing the examination of multiple meanings of multiculturalism around the world, to make comparisons across different countries, and to form predictions concerning the success of multicultural policies.

## Note

1 We also explored whether our MCI-r dimensions would relate to participants’ perceptions of COVID-19 having had negative consequences on their well-being. No significant correlation was revealed with any of the MCI-r’s subscales.
